# Characterization of the transcripts of human cytomegalovirus UL144

**DOI:** 10.1186/1743-422X-8-299

**Published:** 2011-06-14

**Authors:** Rong He, Yanping Ma, Ying Qi, Ning Wang, Mali Li, Yaohua Ji, Zhengrong Sun, Shujuan Jiang, Qiang Ruan

**Affiliations:** 1 Clinical Genetics Department, The Affiliated Shengjing Hospital, China Medical University, 110004 Shenyang, Liaoning of China; 2 Virus Laboratory, The Affiliated Shengjing Hospital, China Medical University, 110004 Shenyang, Liaoning of China

**Keywords:** cytomegalovirus, transcription, UL144

## Abstract

**Background:**

The genome of human cytomegalovirus (HCMV) has been studied extensively, particularly in the UL/b' region. In this study, transcripts of one of the UL/b' genes, UL144, were identified in 3 HCMV isolates obtained from urine samples of congenitally infected infants.

**Methods:**

Northern blot hybridization, cDNA library screening, and RACE-PCR were used.

**Results:**

We identified at least 4 differentially regulated 3'-coterminal transcripts of UL144 in infected cells of 1,300, 1,600, 1,700, and 3,500 nucleotides (nt). The 1600 nt transcript was the major form of UL144 mRNA. The largest transcript initiated from the region within the UL141 open reading frame (ORF) and included UL141, UL142, UL143, UL144, and UL145 ORFs.

**Conclusions:**

These findings reveal the complex nature of the transcription of the UL144 gene in clinical isolates.

## Background

Cytomegaloviruses (CMVs) are members of the subfamily Betaherpesvirinae of the family Herpesviridae. Human cytomegalovirus (HCMV) is a common cause of congenital viral infections and a frequent opportunistic pathogen in transplant recipients and AIDS patients [1-3]. The relative genomic complexity of HCMV is mirrored by its biological characteristics, as the most relevant cellular reservoirs of the latent virus and its sites for permissive replication have not been conclusively established and its pathogenesis is not well understood. Like other CMVs, HCMV has a very specific host range, but within a permissive host it enters and replicates in a wide variety of cell types [3,4].

The HCMV genome consists of 230 to 235 kb of double stranded DNA and more than 160 predicted open reading frames (ORFs) [5-8]. The overall nucleotide sequence of strains isolated from unrelated sources is relatively conserved. However, the sequences of specific ORFs can be highly variable, including RL6, RL12, UL4, UL18, UL55 (gB), UL73 (gN), UL74 (gO), UL139, UL144, and UL146 [5,9-18].

The HCMV UL144 ORF encodes a homologue of the herpesvirus entry mediator (HVEM) [19], which is a member of the tumor necrosis factor receptor (TNFR) superfamily and contributes to the ability of HCMV to escape immune clearance. Although the UL144 gene has been well characterized at the DNA and protein levels, no detailed analysis of its mRNA transcripts has been reported. In the current study, we characterized the transcripts of the UL144 gene in 3 clinical strains.

## Methods

### Cells and virus

Human embryonic lung fibroblast (HELF) cells were grown in Dulbecco's modified Eagle's medium supplemented with 10% (vol/vol) fetal calf serum. Three HCMV clinical isolates, named H, Xu, and Ch, were categorized as Group C, A, and B, according to the schema of Lurain *et al *[9]. The isolates were derived from urine samples of three congenitally HCMV-infected children in the Pediatrics Department at the Affiliated Shengjing Hospital of China Medical University. All samples were collected with the permission of the infants' parents and the research had obtained the approval of the Hospital Ethical Committee. HELF cells were infected with the virus at a multiplicity of infection of approximately 10 plaque-forming units (PFU) per cell. Virus DNA of HCMV isolate H was extracted as previously described [20] and its UL/b' region was sequenced using a previously described shotgun sequencing method [21].

### RNA preparation

Total RNA was extracted from uninfected and HCMV-infected HELF cells using a standard guanidium isothyocyanate and phenol:chloroform method [22]. The extracts were treated with DNase I (Ambion, USA). Immediate early (IE) RNA was prepared in the presence of 100 *μ*g/mL cycloheximide (Sigma, USA) at 24 hours post-infection (hpi), early (E) RNA was extracted in the presence of 100 *μ*g/mL phosphonoacetic acid (Sigma) at 48 hpi, late RNA was extracted in the absence of blocking agents at 96 hpi, and control RNA was extracted from uninfected cells [23].

### Screening of the cDNA library

A cDNA library of the HCMV clinical strain H had been previously constructed using the pBluescript II SK vector [24]. The primary library consisted of 1.12 × 10^6 ^recombinant clones/mL. Nearly 95% of the vectors contained inserts, as determined by blue-white plaque screening on NZY agar plates containing 5-bromo-4-chloro-3-indolyl-β-D-galactopyranoside (X-Gal) and isopropyl-β-D-thio-galactoside (IPTG). The average length of the inserts was 1.2 kb as determined by PCR with M13 primers and the Gel Image System.

A total of 4,000 single clones were randomly picked and inoculated in LB medium. In order to identify gene-specific clones, aliquots of the 4,000 cultures were mixed step by step as follows: a mixture of every 10 individual clones was designated as the first grade of colonies, a mixture of every 10 of the first grade of colonies as the second grade of colonies, and a mixture of every 10 of the second grade of colonies as the third grade of colonies, equivalent to 1000 clones. Each grade of the colonies was inoculated into fresh medium. Cells were lysed using a cell lysis buffer (50 mM Tris-HCl, pH 6.8, 15 mM NaCl, 5 mM EDTA, 0.5% NP-40). The UL144-containing cDNA clones were identified by PCR using a set of primers, 144For2 and 144Rev1 (Table 1), covering the whole UL144 ORF, and each of the DNA preparations from the third, second, and first grade of colonies and single clones were used as templates, sequentially. The PCR conditions were an initial denaturation step at 94°C for 5 min, 30 cycles of 94°C for 30 sec, 50°C for 30 sec, and 72°C for 1 min, followed by a final elongation step at 72°C for 5 min. Identified clones were sequenced using the T7 primer of the pBluescript II SK vector with an ABI PRISM 3730 DNA analyzer. Only sequences with additional polyadenylated residues at their 3' terminal ends were included as entire mRNA sequences.

**Table 1:** Primers used for PCR amplification, Northern-blot and selection of cDNA clones.

Name of Primers	Sequence	Position in UL144 ORF	Position in GQ981646
144Rev1	5'-ttacagggtgcggtagaaaatt-3	510-531	12093-12114

144Rev2	5'-acaaccaggctagagtatgacgacc-3'	388-412	11971-11995

144For1	5'-gtgaagatggctgactatcct-3'	-182~-162	11401-11421

144For2	5'-atgaagcctctggtgatgct-3'	1-20	11584-11603

144For3	5'-aaaatgtgtaagcccgatga-3'	61-80	11644-11663

144Prob	5'-taatacgactcactatagggttacagggtgcggtagaaaattttg-3'	507-531	12090-12114

A complete list of all primers is provided in Table 1.

### Northern blot hybridization

RNA was prepared from uninfected cells and cells infected with HCMV clinical isolates H, Ch, and Xu at IE, E, and L phases. RNA was analyzed by Northern blot to identify the transcripts of UL144.

Five micrograms of RNA was size-fractionated in a 1.2% denaturing agarose gel and transferred to a membrane (N-Hybond, Amersham Biosciences) by capillary blotting. A UL144 gene-specific probe was labeled with digoxigenin-dUTP, hybridized to the membrane, and detected by its NBT/BCIP chromogenic signal according to the manufacturer's instructions (DIG Northern starter kit, Roche Diagnostics, Mannheim, Germany). RNA representing the noncoding strand was generated using T7 RNA polymerase and labeled by incorporation of digoxigenin-dUTP. Three probes were produced according to the different sequences of isolates H, Ch, and Xu using the primers 144For2 and 144Prob (Table 1). The labeled probes were antisense to the 531 bp of the complete UL144 protein coding sequences (nucleotides 11584-12114, GQ981646 as reference) of the three isolates. Hybridization to the RNA blots was carried out at 55°C overnight. The blots were visualized by the addition of an anti-digoxigenin antibody labeled with alkaline phosphatase followed by the addition of the substrate of 5-bromo-4-chloro-3-indolyl phosphate (BCIP) and nitroblue tetrazolium (NBT). DIG-labeled RNA Molecular Weight Marker I 0.31-6.95 kb (Roche Diagnostics) was used to estimate the size of the bands by the logarithmic relationship between molecular weight and distance migrated.

*5' RACE *To define the 5' ends of transcripts in the UL144 intergenic region, RNA preparations of the isolates H, Ch, and Xu were analyzed by 5' RACE, which was carried out with the 5'-Full RACE kit (Takara, Japan) according to the manufacturer's instructions. Briefly, the RNA preparation was treated with calf intestine alkaline phosphatase (CIAP, 4.8 U/μg RNA) for 1 h at 50°C. Then the purified RNA was treated with tobacco acid pyrophosphorylase (TAP, 0.25 U/μg RNA) for 1 h at 37°C. The CIAP/TAP-treated RNA was ligated to the supplied 5' RACE adaptor (1 μL of 15 μM adaptor/2 μg RNA) with bacteriophage T4 RNA ligase (20 U/μg RNA) for 1 h at 16°C. Reverse transcription was carried out at 42°C for 1 h with 5 U M-MLV reverse transcriptase and 5 μM of random 9-mer primers. Control reactions were carried out in parallel using RNA treated without M-MLV [M-MLV(-)]or TAP [TAP(-)]. All reactions were performed in the presence of 10 U RNase Inhibitor. cDNA was amplified by nested PCR using 1.25 U LA Taq DNA polymerase (Takara) in a 50 μL reaction mixture including LA PCR buffer (50 mM KCl, 10 mM Tris-HCl, pH 8.3), 1.5 mM MgCl_2_, and 2.5 mM of each of the four deoxynucleoside triphosphates. The primers used in the primary PCR reaction were the 5' RACE outer primer and 144Rev1 (see Table 1 for primer coordinates within the H isolate sequence) and those in the second PCR reaction were the 5' RACE inner primer and 144Rev2. The PCR conditions included an initial denaturation step at 94°C for 3 min, 20-25 cycles of 94°C for 30 sec, 55°C for 30 sec, and 72°C for 3 min, followed by a final extension step at 72°C for 10 min.

*3' RACE *3' RACE was carried out with the 3'-Full RACE Core Set Ver. 2.0 (Takara) following the manufacturer's instructions. Briefly, 500 ng of RNA was used as a template to synthesize cDNA with AMV Reverse transcriptase for 30 min at 50°C, 5 min at 99°C and 5 min at 5°C. The external reverse primer oligo dT-adaptor primer with a poly (T) tract was used to prime cDNA synthesis. cDNA of the 3 clinical isolates, including isolates H, Ch, and Xu, was then amplified by nested PCR with an adaptor primer and the external forward primer 144For2 and the inner forward primer 144For3.

*RT-PCR *RT-PCR was carried out using the RNA PCR Kit (AMV) Ver. 3.0 (Takara) according to the manufacturer's instructions. Reverse transcription was performed at 42°C for 1 h with 200 U of AMV Reverse Transcriptase (Takara) and 25 mM of oligo dT-adaptor primer. PCR was conducted using 10 μL of the synthesized cDNA and primers 144For1 and 144Rev2 (Table 1) with Ex Taq DNA polymerase. The PCR conditions for all the amplification reactions included an initial incubation at 94°C for 3 min, followed by 40 cycles of denaturation at 94°C for 40 sec, annealing at 50°C for 40 sec, and extension at 72°C for 2 min, with a final incubation at 72°C for10 min. Control reactions using RNA treated without AMV [AMV (-)] and water as templates were included in each set of PCR amplifications.

### Cloning and sequencing

RACE and RT-PCR products were separated by electrophoresis on a 2% agarose gel and visualized with ethidium bromide. Bands were gel purified and the recovered fragments were ligated into a TA cloning vector, pCR (Invitrogen, USA). Inserts were sequenced by the dideoxynucleotide method with Sequenase (Invitrogen) and M13For and M13Rev primers using an Applied Biosystems automated sequencer.

## Results

### Identification of UL144 transcripts by cDNA library screening

Twenty-two distinct clones were identified from the cDNA library and all were sequenced successfully. The 3' ends of the sequences occurred within a 3-nucleotide (nt) window extending from nt 12,844-12,846 (GQ981646 as reference). Five groups of UL144 transcripts were derived from the 22 cDNA sequences according to their different 5' ends. Among the 22 cDNA sequences, 15 (named as C1) transcripts were 1565-1612 nt in length with 5' ends at nt 11,276-11,278 (GQ981646 as reference) and different lengths of poly A tails, 4 (named as C3) were 1317 nt with their 5' ends at nt 11,528, and 1 (named as C2) was 1775 nt with its 5' end at nt 11,091. The other two transcripts were 1230 nt and 1161 nt in length (named as C4 and C5, respectively) and started at nt 11,635 and 11,704, respectively, indicating that both of the transcripts initiate within the UL144 ORF.

### Mapping the 5' end of UL144 transcript using 5' RACE-PCR

**Figure 1:**
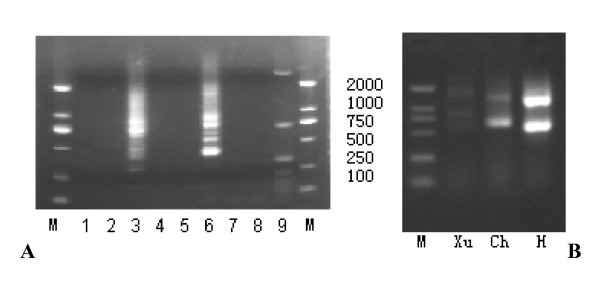
**Characterizations of the HCMV UL144 transcripts by 5'-RACE-PCR and 3'-RACE-PCR analyses**. 5'-RACE-PCR and 3'-RACE-PCR were performed on first-strand cDNA prepared from total RNA from HELF cells infected with HCMV for 96 h. Strains Xu, Ch, H were analyzed respectively. **A **5'-RACE-PCR analysis was performed using the gene specific primers of 144Rev1 and 144Rev2 and the templates prepared with (lanes3, 6 and 9) or without reverse transcriptase (lane1, 4 and 7) during the first strand cDNA synthesis or without tobacco acid pyrophosphorylase (lane2, 5 and 8) during RNA treatment of the isolates. Strains Xu (lanes1-3), Ch (lanes4-6), H (lanes7-9) were analyzed respectively. **B **3'-RACE-PCR analysis was performed on first-strand cDNA using the external forward primer 144For2 and the inner forward primer 144For3 by nested PCR together with oligo(dt)17-adaptor. Molecular standards are labeled 'M'. The positions of DL2000 molecular markers are indicated.

Eight products of approximately 100, 150, 200, 350, 465, 720, 860, and 2693 bp were obtained from the 5' RACE-PCR (Figure 1A). Sequencing of the products revealed that they had different 5' ends. The 5' end of the 2500 bp product occurred at nt 9,303, which is located within the UL141 ORF. This product was obtained in two separate experiments using RNA preparations of isolate H, but was not found in the RNA of the other two isolates. The 5' ends of the sequences of the 900, 700, and 500 bp products were at nt 11,100, 11,276, and 11,528, respectively, and the three products were obtained from the RNA of all three isolates. The 5' ends of the sequences of the four products 350 bp in size and smaller were at nt 11,637, 11,822, 11,878 and 11,935 respectively.

### Mapping the 3' end of UL144 transcript using 3' RACE-PCR

As shown in Figure 1B, 3' RACE-PCR yielded two products of 1.3 kb and 500 bp in length. Five UL144-specific clones with the 1.3 kb band from isolates H (1), Ch (1), and Xu (3) were sequenced. All the sequences terminated at nt 12,845, except for one from isolate Xu, which terminated at nt 12,844. Sequencing of the 500 bp band revealed it to be a nonspecific product.

### Identification of UL144 transcripts by Northern blot

**Figure 2:**
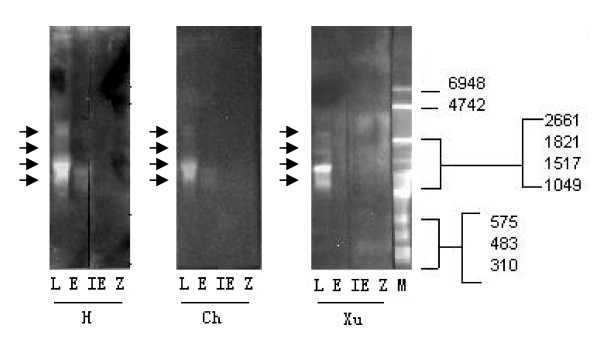
**Northern blot analyses of UL144 transcripts**. RNA preparations from HCMV clinical strains H, Ch and Xu in immediately early (IE), early (E) and late (L) kinetics classes were used in this analysis. RNA from non-infected HELF cells (Z) was used as a negative control. The Northern blot was performed with three different probes, produced according to the sequences of the three different isolates using 144For2 and 144Prob primers. The four UL144 transcripts are indicated by arrows. The positions of RNA size markers are indicated.

Northern blot revealed the hybridization of the probe to 4 bands in RNA preparations from the L phase of viral replication in isolate H (Figure 2). The major band was approximately 1600 nt and it appeared repeatedly. The three fainter bands were approximately 1300, 3000, and 3500 nt. Similar results were obtained for isolate Ch. The four bands in RNA preparations from the L phase of viral replication in isolate Xu were approximately 1300, 1600, 2000, and 3500 nt. The RNA from uninfected cells was negative in all the hybridization experiments. No bands were found in the RNA preparations of the IE or E phases from any of the three strains.

## Discussion

The UL144 gene is located in the UL/b' region of the HCMV genome, which contains approximately 20 kb of DNA encoding at least 19 ORFs but is dispensable for growth *in vitro *[25]. The UL144 ORF encodes a structural homologue of the herpesvirus entry mediator (HVEM) [9,26], a member of the TNFR superfamily, but it does not bind any known TNF-related ligands [9,26,27]. It negatively regulates Th1 cells by binding to the B- and T-cell lymphocyte activator BTLA [28], mimicking the inhibitory co-signaling function of HVEM. Like LMP-1 of the Epstein-Barr virus, UL144 activates TNFR-activated factor 6 (TRAF6), leading to NF-κB (nuclear factor kappa-light-chain-enhancer of activated B cells) activation in early infection [27]. It induces expression of the macrophage-derived chemokine (MDC) [27,29,30], also termed CCL22 [chemokine (C-C motif) ligand 22], which attracts Th2 and regulatory T cells. Taken together, it is possible that upregulation of CCL22 by the UL144 protein may help HCMV evade immune surveillance, resulting in increased viral spread and dissemination. There is significant strain-specific sequence variability in the UL144 ORF [9,18,26,31,32] and these UL144 genotypes are associated with congenital infection outcome [31,33]. However, no data has been reported thus far on the UL144 mRNA structure.

**Figure 3:**
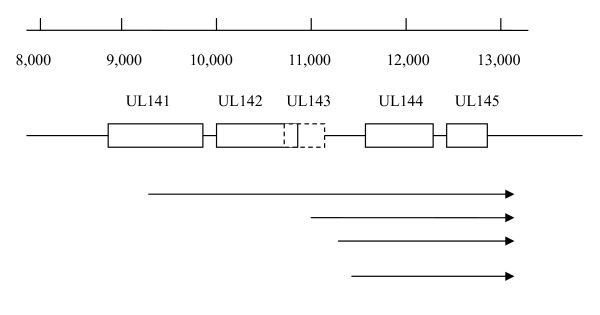
**Summary of UL144 mapping results**. Transcripts of the HCMV UL144 gene demonstrated in this study are indicated. All of the transcripts share the same 3' end at nt 12844-12846. Nucleotide numbers are based on the UL/b' region sequence of isolate H (GenBank accession number GQ981646).

In this study, we provide evidence for the existence of at least 4 differentially regulated UL144 transcripts of 1,300, 1,600, 1,700, and 3,500 nt (Table 2, Figure 3). The 5' ends of the 1,300, 1,600, and 1,700 nt transcripts are within the region between the UL143 and UL144 ORFs and the transcripts all contain the same 2 ORFs of UL144 and UL145. The 1,600 nt transcript appears to be the major form since its presence was confirmed by all of the methods used in this study, including Northern blot hybridization, RACE-PCR, and cDNA library screening. At the same time, this transcript is the most abundant based on its prevalence in the cDNA library (15 of 22 cDNA clones) and the strength of its band in the Northern blot assay. Potential TATA and CCAAT promoter elements at 29 and 70 bp upstream of its initiation site, respectively, further support the existence of this transcript. The 1,300 nt and 1,700 nt transcripts were not as prevalent as the 1,600 nt transcript. The 1,300 nt transcript was found in 4 of 22 clones from the cDNA library and while its genomic sequence contains a potential TATA promoter element 13 bases upstream of its initiation site, it does not contain a CAT box. The transcript of 1,700 nt was present in only 1 of the 22 specific cDNA clones and was not detected by Northern blot, most likely due to its low prevalence. It has potential TATTA and CACAT promoter elements at 50 and 90 bases upstream its initiation site, respectively. The largest transcript, 3,500 nt in length, initiates from within the UL141 ORF and includes UL142, UL143, UL144, UL145, and part of UL141 ORFs. This transcript was found by Northern blot only at long exposure times and its initiation site was demonstrated in two separate 5' RACE experiments using RNA preparations from isolate H. A potential promoter (TTACTTTTAA) and a "CCAAAT" were found at -15 and -29 of its initiation site, respectively. However, the presence of the 3500 nt transcript was not confirmed in the cDNA library. The long length of the transcript may have prevented its ligation during the construction of the cDNA library.

**Table 2:** Transcripts of the HCMV UL144 gene demonstrated in this study.

Transcripts	The position of 5' end of the transcript	Isolates in which transcripts were identified	Amounts. of cDNA clones from the library	Strength of reaction in Northern blot	Position and forms of the TATA-box	Position and forms of the CAT-box
1,600 nt	11276	H,CH,XU	15	++++	11247-11250,TATA	11206-11210,CCAAT

1,300 nt	11528	H,CH,XU	4	++	11515-11519.TATTA	-

3,500 nt	9303	H	0	+	9288-9297.TTACTTTTAA	9274-9279. CCAAAT

1,700 nt	11091	H,CH,XU	1	-	11040-11044. TATTA	11001-11005. CACAT

Note: all of the transcripts share the same 3' end at nt 12844-12846. Nucleotide numbers are based on the UL/b' region sequence of isolate H (GenBank accession number GQ981646).

In this study, all 22 UL144-specific cDNA clones terminated within a 3 nt window located 100 bp downstream of the UL145 gene. The single poly(A) signal, which is downstream of the UL145 stop codon, supports the possibility of a common 3' terminal end for these transcripts. The 3' terminal ends of the transcripts were confirmed by 3' RACE using RNA from all the three isolates. The presence of 3' coterminal transcripts around the UL144 region is similar to that found in other genomic regions of HCMV, including the UL93-UL99 ORFs [34], UL146-UL132 ORFs [35], and M73 and M73.5 genes [36]. It is possible that their different 5' non-coding sequences may have regulatory functions, warranting further investigation.

We obtained some small RNA fragments in our cDNA library and by 5' RACE that initiates inside the UL144 ORF. These RNA fragments are most likely products from incomplete transcription. Transcripts of approximately 3,000 nt in the RNA of isolates H and Ch and approximately 2,000 nt in the RNA of isolate Xu were present in our Northern blots but were not confirmed by cDNA library screening or 5' RACE-PCR, indicating that they may be due to non-specific hybridization.

## Conclusions

In this study, we have demonstrated that the transcripts of UL144 have a complex transcriptional pattern of overlapping transcripts that differ at their 5' initiation sites in different UL144 genotype isolates. At least 4 transcripts were identified in the 3 clinical isolates studied. These findings reveal the complex nature of the transcription of the UL144 gene in clinical isolates.

## List of abbreviations

HCMV: human cytomegalovirus; HVEM: herpesvirus entry mediator; ORF: open reading frame; TNFR: tumor necrosis factor receptor; HELF: Human embryonic lung fibroblast; PFU: plaque forming units; IE: immediate early; X-Gal: 5-bromo-4-chloro-3-indolyl-β-D-galactopyranoside; IPTG: isopropyl-β-D-thio-galactoside; TRAF6: TNFR-activated factor 6; NF-κB: nuclear factor kappa-light-chain-enhancer of activated B cells; MDC: macrophage-derived chemokine; CCL22: chemokine (C-C motif) ligand 22.

## Competing interests

The authors declare that they have no competing interests.

## Authors' contributions

RH carried out primer design, PCR and sequence analysis. As the corresponding author, QR conceived the experimental design and participated in revising the manuscript. NW and MLL carried out virus preparation and cell culture. YQ and YPM carried out RNA isolation and mRNA purification. ZRS and YHJ carried out plasmid construction. SJJ carried out Northern blot analysis. All authors have read and approved the final manuscript.
